# Twenty years after Erich Muhe: Persisting controversies with the gold standard of laparoscopic cholecystectomy

**DOI:** 10.4103/0972-9941.26646

**Published:** 2006-06

**Authors:** Kalpesh Jani, P S Rajan, K Sendhilkumar, C Palanivelu

**Affiliations:** Departments of Gem Hospital, 45A, Pankaja Mill Road, Ramanathapuram, Coimbatore - 641045, India

**Keywords:** Controversies, Erich Muhe, laparoscopic cholecystectomy

## Abstract

This review article is a tribute to the genius of Professor Erich Muhe, a man ahead of his times. We trace the development of laparoscopic cholecystectomy and detail the tribulations faced by Muhe. On the occasion of the twentieth anniversary of the first laparoscopic cholecystectomy, we take another look at some of the controversies surrounding this gold standard in the management of gallbladder disease

During the early 1980s, news of Semm's laparoscopic appendectomy was rippling through German medical circles. A young German surgeon, Erich Muhe, working in the Department of Surgery of the Boblingen Hospital, was fascinated by Semm's technique and Lukichev's method of minimally invasive cholecystostomy. He developed the idea of laparoscopic removal of gallstones. In 1984, Muhe had already worked out the finer details of an operative laparoscope, calling it the “Galloscope”. On September 12^th^ 1985, Prof. Erich Muhe of Boeblingen, Germany, carried out the first laparoscopic cholecystectomy. Later, he modified his technique and operated through a trocar sleeve. Finally, he designed an “open laparoscope” with a circular light. By March 1987, Muhe had conducted 97 endoscopic gallbladder removals. He published information about his technique at the Congress of the German Surgical Society (April 1986) and at other surgical meetings in Germany. After reporting that he had performed the first laparoscopic cholecystectomy in The German Surgical Society meeting in 1986, he was severely derided and criticized.[1] It took his fraternity almost six years in 1992 to recognize his contribution and he received their highest award, the German Surgical Society Anniversary Award. Across the ocean, his more fortunate contemporaries were reaping rich rewards and recognition. In 1990, in Atlanta, at the Society of American Gastrointestinal Surgeons (SAGES) Convention, Perissat, Berci, Cuschieri, Dubois and Mouret were recognized by SAGES for performing early laparoscopic cholecystectomies, but Muhe was not. However, in 1999, he was recognized by SAGES for having performed the first laparoscopic cholecystectomy. SAGES invited Muhe to present the Karl Storz Lecture. In Muhe's presentation titled “The First Laparoscopic Cholecystectomy,” which he gave in March 1999 in San Antonio, Texas, he described the first procedure. Finally, Muhe had received the acclaim that he deserved for his pioneering work.[2]

Across the Atlantic, Reddick and Olsen did pioneering work in the field.[3,4] Laparoscopic cholecystectomy has now undisputably become the gold standard for the surgical management of gallstone disease. On the occasion of the twentieth anniversary of the pathbreaking event in the field of surgery, we take a look at the controversies still dogging this procedure, with a review of the concerned literature.

We have concentrated on the following topics, which as per available literature, have not been accepted as standard aspects of this procedure or are construed as being controversial:

1. Prophylactic laparoscopic cholecystectomy.
2. Role of laparoscopic cholecystectomy in acute cholecystitis.
3. Intra-operative cholangiography and the management of associated CBD stones.
4. Different variants of laparoscopic cholecystectomy, including gasless laparoscopic cholecystectomy.
5. Occult gall bladder cancer.
6. Complications following laparoscopic cholecystectomy, including spillage of gall stones.
7. Comparison of laparoscopic cholecystectomy to open cholecystectomy.

## PROPHYLACTIC LAPAROSCOPIC CHOLECYSTECTOMY

Since the performance of laparoscopic cholecystectomy in 1985, its safety and efficacy has been proven by several large studies.[5–9] However, in the early 90s, it was found that the number of laparoscopic cholecystectomies being performed had dramatically increased.[10,11] Several reasons have been hypothesized for this-first, it may be due to changing selection criteria for surgical treatment of gallstones. Second, surgery may have been done for asymptomatic gallstones. Third, patients with moderate symptoms who refused the (open) operation in the past, may now be more willing to undergo a laparoscopic cholecystectomy. Finally, it may be due in part, to procedures performed on a large pool of procrastinating, mildly symptomatic patients. However, if surgeons are performing laparoscopy on asymptomatic patients with gallstones, then these rates may well be sustained. Such a broadening of indications for gallbladder surgery is of concern. Any broadening of indications for gallbladder surgery, also has significant implications for health care costs and the use of health care resources.

There were a few studies that concluded that there was a role for prophylactic cholecystectomy for all patients with asymptomatic gallstones.[12–14]

The practice of prophylactic cholecystectomy has since been challenged by several studies.[15–18] Gracie and Ransohoff[16] followed a cohort of 123 university faculty members with asymptomatic gallstones and found that the 15-year cumulative probability of biliary symptoms or complications was only 18 percent, with no deaths. McSherry *et al*[15] followed 135 asymptomatic patients with gallstones for a mean of nearly five years and found that symptoms developed in only 10 percent and that only 7 percent required surgery. On the basis of risk-benefit analysis, prophylactic cholecystectomy expectancy[17] is not recommended for most asymptomatic patients.[18,19]

Prophylactic cholecystectomy for gallstones has been recommended in specific groups such as children, because symptoms develop in almost all patients.[20] It has also been recommended in patients with gallstones and sickle cell disease, because the symptoms of gallstones can mimic those of sickle cell crisis and selective cholecystectomy is much safer than emergency cholecystectomy in this group.[21] Incidental cholecystectomy for cholelithiasis is often performed concomitantly with surgery for morbid obesity, in view of the high incidence of symptomatic gallstones during rapid weight loss.[22] Some surgeons have recommended incidental cholecystectomy for cholelithiasis in patients undergoing other abdominal surgery.[19]

Prophylactic cholecystectomy is also recommended in certain high-risk groups to prevent gallbladder cancer. Native Americans are especially at high risk of gallbladder cancer, particularly if they have gallstones, in which case the risk is 3 to 5 percent.[23] In the general population, 80 percent of the patients with gallbladder cancer have gallstones, with an especially high risk with longstanding stones or stones greater than 3 cm in diameter.[24] Gallbladder cancer also occurs in half or more of the patients with a calcified gallbladder wall or “porcelain” gallbladder.[25] Hence, laparoscopic cholecystectomy has been recommended for patient subgroups which have a high prevalence of gallbladder cancer.[26]

Until recently, prophylactic cholecystectomy was recommended for diabetic patients with gallstones. Older studies had shown that people with diabetes mellitus have an increased risk of acute cholecystitis and increased mortality with emergency cholecystectomy. Recent studies show that diabetic patients have increased operative risk with selective, as well as emergency gallbladder surgery.[27] This increased risk is related to cardiovascular disease and other coexisting conditions, rather than to diabetes mellitus itself.[27,28]

In summary, the various indications for performing laparoscopic cholecystectomy in patients with asymptomatic gallstones are:[19–25] 1. life expectancy > 20 years (young children); 2. calculi > 2 cm in diameter; 3. calculi < 3 mm and a patent cystic duct; 4. radiopaque calculi; 5. polyps in the gallbladder (GB); 6. nonfunctioning GB; 7. calcified (“porcelain”) GB; 8. concomitant diabetes, cirrhosis or chronic hemolytic anemia; 9. those that are candidates for kidney or heart transplantation and those with underling degenerative diseases, that are more likely to develop severe complication of cholelithiasis; 10. women < 60 years/ selected women of childbearing age; 11. individuals in geographic regions with a high prevalence of GB cancer; 12. patients undergoing other upper abdominal surgery; 13. in association with surgery for morbid obesity.

However, these indications have still not been standardized and further studies have to be done to formulate universal guidelines for the management of asymptomatic gallstones.[26,29,30]

There is a better agreement amongst surgeons regarding the laparoscopic management of polypoid lesions of gallbladder, in absence of gallstones. Adenomyomatosis of the gallbladder wall, gallbladder polps associated with biliary pain, asymptomatic polyps larger than 1 cm. in size and asymptomatic polyps in patients aged 50 years or older, are recommended to undergo laparoscopic cholecystectomy.[31–34]

## LAPAROSCOPIC CHOLECYSTECTOMY IN ACUTE CHOLECYSTITIS

In the early days, after the first laparoscopic cholecystectomy, acute cholecystitis was considered as an absolute and later, a relative contraindication for this surgery.[35] It was believed that the risk of morbidity, especially common bile duct injuries, was higher in the setting of acute cholecystitis.[36] However, now it has been established that laparoscopic cholecystectomy can be safely performed by experienced surgeons in the setting of acute cholecystitis.[37–39] Even the next controversy to crop up, viz. the timing of surgery, seems to have been resolved with most of the surgeons favoring early intervention rather than delayed surgery.[40–44]

### Intra-operative cholangiogram and the management of associated choledocholithiasis

There is some confusion regarding the correct management of patients posted for laparoscopic cholecystectomy, with suspected or proven choledocholithiasis.[45–49] The rate of coexisting common bile duct stones in patients undergoing cholecystectomy for cholelithiasis is approximately 7–20%.[45,47] It is generally accepted that bile duct stones should be removed (even if asymptomatic), because they may be associated with severe complications such as pancreatitis and cholangitis. Routine preoperative ERCP may not be recommended, due to the low percentage of coexisting choledocholithiasis, a large number of negative investigations and a small but significant risk of associated morbidity and high additional costs. Postoperative ERCP could reduce the number of unnecessary interventions and the majority of retained stones and postoperative leakages can be treated, although a second operation is required in case of failure. The development of more reliable predictors of CBD stones, based on the patient's clinical, biochemical and ultrasound (US) presentations, could allow a more appropriate use of preoperative ERCP (or EUS, MRCP). Several authors have constructed complicated scoring systems[46,47,50] to predict the presence of CBD stones. However, certain basic clinical and sonographic features may lead to a suspicion of the presence of CBD stones. These are:[51]

- Common bile duct dilated (> 8–10 mm on ultrasound).
- Recent abnormal levels of liver enzymes or bilirubin.
- History of acute pancreatitis.
- History of obstructive jaundice.
- History of cholangitis.

These patients can be subjected to pre-operative ERCP. If the surgeon is sufficiently skilled, he can proceed for intra-operative cholangiography and laparoscopic CBD exploration.

Cotton[52] has proposed that the main indications of preoperative ERCP were the following: positive predictive factors for CBD stones, the expertise of the endoscopist and the pressure for laparoscopic intervention as opposed to open surgery. He identified low, medium and high-risk patients for CBD stones based on the clinical features, liver function tests and ductal dilation on ultrasound. According to his conclusions, preoperative ERCP was not indicated for low-risk patients, while it must absolutely be performed in the high-risk group. As for the medium risk group, preoperative ERCP was only indicated, if the local endoscopist was mediocre. With an experienced endoscopist, preoperative intervention should be avoided and ERCP is to be performed only after the surgery, if the need arises. If available, MRCP can be a good alternative to ERCP. In case of a highly qualified endoscopist, ERCP should be postponed until the surgery can be considered.[53] If the surgeon is technically confident, he can consider intraoperative cholangiography and laparoscopic CBD exploration.

### Other variants of laparoscopic cholecystectomy including ‘gasless laparoscopic cholecystectomy’

One of the major drawbacks of laparoscopic surgery has been due to the carbon dioxide pneumoperitoneum. Induction and maintenance of carbon dioxide pneumoperitoneum can have severe physiological disturbances.[54–56] Although rare, the potential complications have stimulated the search for alternative methods of obtaining the intra-abdominal space necessary for laparoscopic surgery. These complications have been the reason pointed out by various authors who use mechanical lifting of the abdominal wall (gasless laparoscopy).[57–60] The mechanical lifting method of the abdominal wall dispensed with gas insufflation, is done by allowing an adequate space to be created in the intra-abdominal region for laparoscopic surgery, based on the traction and subsequent elevation of the abdominal wall. Gasless laparoscopy has been found to be easy and risk- free. It is especially useful when operating on critical patients with a cardiorespiratory problem, who would benefit most from laparoscopic surgery due to the reduced trauma and advantages for recovery.

In cases of difficult cholecystectomies, various approaches have been advocated, like the ‘dome down’ approach[61] or tape ligature of cystic duct and fundus down approach.[62] To increase the acceptability of the procedure, mini-laparoscopic cholecystectomy using all 5 mm trocars[63] or 2–3 mm trocars[64,65] has been performed. Several centers carry out the procedure on an outpatient basis.[66] Absorbable clips have been used, but have not been found to be advantageous.[67] Harmonic scalpel has been used as the sole instrument for dividing the cystic duct and artery (‘clipless laparoscopic cholecystectomy’).[68] Its use has been found to decrease the incidence of gallbladder perforation and decreases the time required for the surgery.[69] A dilated cystic duct may be difficult to control with a clip. A pre-tied loop or an Endo-GIA stapler can be used for the same.[70] A combined method of endoscopic sphincterotomy with common bile duct stone extraction and laparoscopic cholecystectomy under general anesthesia, for a single-session treatment of patients with gallstones with simultaneous CBD stones is described, - the so called “rendez-vous” technique.[71] Various innovative techniques have been adopted for clear identification of the common bile duct, including laparoscopic intracorporeal ultrasound cystic duct length measurement,[72] filling the extra-hepatic biliary system with methylene blue[73] and using cold light illumination of the extrahepatic biliary system (light cholangiography LCP) by leading an optical fiber into the common duct with a duodenoscope at the time of LC.[74] However, Strasberg has recommended that by obtaining the “critical view of safety”, there are two and only two structures entering the gallbladder, which is otherwise still attached only by the upper part of the liver bed. The triangle of Calot is dissected free of all tissue, except for cystic duct and artery and the base of the liver bed is exposed. When this view is achieved, the two structures entering the gallbladder can only be the cystic duct and artery. It is not necessary to see the common bile duct.[75,76]

The future direction lies in the development of robotic surgery.[77]

Today, just a few years after the first systems reached the market, the feasibility of various laparoscopic procedures including transcontinental robot-assisted remote surgery (telesurgery) has been reported.[78,79] There are now several reports documenting the safety and feasibility of robotic surgery in humans.[80–82] Even though there are no clinical trials available for verifying the advantages of robotic over conventional surgery, robots have the potential to revolutionize the way surgery is performed.[78] Robotic laparoscopic cholecystectomy offers the advantage of surgeon comfort, elimination of surgeon tremor and improved imaging and increased degrees of freedom of the operative instruments, but has the disadvantage of being more time-consuming because of slower performed actions.[83,84]

### Management of occult gallbladder cancer

A dilemma facing the laparoscopic surgeon is how to deal with occult gallbladder cancer. If malignancy is suspected pre-operatively, the course is clear – open laparotomy is the norm. However, when cancer is detected in the post-operative specimen following laparoscopic cholecystectomy, the consensus is that in stage Tis or T1, laparoscopic cholecystectomy is sufficient. In stage T2 and T3, a repeat operation with liver bed resection and lymphadenectomy has to be performed.[85,86] The impact of laparoscopic cholecystectomy on the long term prognosis of patients with gallbladder cancer is controversial, with some studies claiming that the long-term prognosis of patients with undiagnosed gallbladder cancer who underwent LC was not worsened by the laparoscopic procedure[87,88] and other studies claiming the reverse.[89] If high resolution ultrasound reveals the slightest suspicion of carcinoma, open cholecystectomy with frozen section should be performed.

### Complications following laparoscopic cholecystectomy, including gallstone spillage

One of the the commonest complication has been cystic duct biliary leak, revealed by post-operative bile leak in the drain tube. It can occur due to injury to the common duct, the right hepatic duct or accessory bile duct. In case of acute inflammation, the clip applied to the cystic duct may become loose once the edema subsides and subsequently slip off. Correct identification of the cystic duct and artery, minimum use of electrocautery in Calot's triangle dissection and appropriate choice of laparoscopic subtotal cholecystectomy, will help in avoiding this complication. In the setting of acute cholecystitis, when proper application of the clip is doubtful, it may be advisable to use a pre-tied suture loop or intra-corporeal suturing to occlude the cystic duct.

In the late 80s and early 90s, a higher incidence of bile duct injury following laparoscopic cholecystectomy had been reported, especially in the setting of acute cholecystitis.[36] However, with adequate experience, this rate has come down. A unique factor predisposing to bile duct injury in laparoscopic cholecystectomy, is that the “infundibular” technique of identifying the cystic duct-gallbladder junction can create an optical illusion called “the hidden cystic duct", resulting in misidentifying the common duct as the cystic duct.[90] The proponents of the “infundibular” technique suggested identifying the junction of the cystic duct and gallbladder by noting the flaring of the infundibulum and the termination of the infundibular flare was considered as the origin of the cystic duct. However, Strasberg pointed out that using this technique, especially in cases with a short cystic duct and with an end-viewing telescope, one was likely to mis-identify the common bile duct as the cystic duct and cause inadvertent injury to the former. He suggested that no tubular structures in the cholecystohepatic triangle should be clipped or divided without obtaining the “critical view of safety". This view can be obtained by dissecting and clearing all fibrofatty tissues between the infundibulum and the liver bed, so that two and only two structures can be seen to be entering the gallbladder, which can only be the cystic duct and artery.[90]

Moreover, accidental injuries to the CBD can also be avoided by confining the dissection to the “safety zone” (cystic duct-gallbladder junction), by staying away from the “danger zone” (cystic duct-CBD junction).[91]

Another complication of laparoscopic cholecystectomy is gallstone spillage. In a meta-analysis of 6 studies comprising 18,280 laparoscopic cholecystectomies, the incidence of gallbladder perforation was 18.3%, that of gallstone spillage was 7.3% and that of unretrieved peritoneal gallstones was 2.4%. The likelihood of a complication when gallstone spillage occurred was 2.3%, which was increased to 7.0% when unretrieved peritoneal gallstones were documented.[92] Unretrieved gallstones can cause a variety of problems, which are summarized in Table 1.[93–119]

**Table 1:** Complications of gallstone spillage

Clinical presentation secondary to gallstone spillage
Infective: Local: Liver abscess. Subhepatic abscess. Retrohepatic abscess. Intra-abdominal abscess. Distant: Retroperitoneal abscess. Loin abscess. Pelvic abscess. Cutaneous complications: Sinus formation. Port site infections. Granuloma formation. Colocutaneous fistula. Mechanical: Intestinal obstruction. Lodgement in distant hernial sacs. Dyspareunia, tenesmus (pelvic migration). Middle colic artery thrombosis. Chest: Empyema, cholelithoptysis. Urinary tract: Excretion, haematuria. Systemic: Septicaemia.

The risk of wound infection following laparoscopic cholecystectomy in literature is less than 1% and the risk of incisional hernia is 0.5%.[120,121] A similar wound problem rate of 0.75% has been reported by Morgenstern for open cholecystectomy.[122] Use of a specimen bag for extraction of the gallbladder and closure of all port sites larger than 8 mm, may help to avoid these complications.

Other complications reported in literature include trocar site bleeding,[123] difficulty in extraction of the gallbladder, bowel injury,[124] injury to the urachus or a Meckel's diverticulum[125,126] and diaphragmatic injury.[127,128]

### Laparoscopic cholecystectomy vs open cholecystectomy

So, the final controversy remains: which is better – open or laparoscopic cholecystectomy? There can be no doubt that with laparoscopic cholecystectomy, the pain felt by the patient is less, overall morbidity is less, recovery is faster, hospital stay is reduced, cosmesis is better and return to work is earlier.[129] As more and more experience is gained, the contraindications to the procedure have shrunk, so that the only absolute contraindications to laparoscopic cholecystectomy are the same as those for open cholecystectomy. There was an initial increased incidence of iatrogenic complications, especially bile duct injury, but even this is gradually coming down. Even after controlling the differences in the clinical characteristics of patients undergoing open as compared with laparoscopic cholecystectomy, such as the greater likelihood that patients undergoing open cholecystectomy would have acute cholecystitis or a common-bile-duct stone, it has been found that the operative mortality was 80 percent lower for laparoscopic cholecystectomy.[130] The results of several large series are summarized in Table 2.

**Table 2:** Comparison of large series

Authors	Total no. of patients (n)	Complications N (%)	Mortality N (%)
Southern surgeons[120]	1518	82 (5.1)	1 (0.07)
Cushieri[16]	1236	20 (1.6)	0 (0)
Daradkeh S[131]	1208	25 (2.1)	1 (0.08)
Wolnerhanssen[132]	3554	71 (2)	0 (0)
Konstadoulakis[133]	5539	162 (2.92)	0 (0)

## CONCLUSION

In face of severe opposition and skepticism, Dr. Erich Muhe developed the basic concept of minimal access for cholecystectomy. The laparoscopic approach has now become the method of choice when cholecystectomy is indicated for benign conditions. The problems have been identified and with improved techniques, laparoscopic cholecystectomy can be performed safely with least morbidity and mortality, similar to or even lower than open cholecystectomy. Large series have documented laparoscopic management with extremely low rates of conversion and bile duct injury. The overall incidence of biliary complications has come down remarkably. One clear advantage of laparoscopic cholecystectomy is the substantial reduction in morbidity related to incision, reduced pain, decreased length of hospital stay and earlier return to work. We dedicate this article to the courage and genius of Dr. Erich Muhe.
